# Incorporating High-Resolution Ultrasound Into Your Practice

**DOI:** 10.1093/asjof/ojad060

**Published:** 2023-06-28

**Authors:** Jeffrey M Kenkel, Christopher C Surek, Bradley Bengtson, Steven Sigalove, Pat Pazmiño

The authors are pleased to present an expert video roundtable on the use of ultrasound in plastic surgery (Video). The discussants include a panel of leading experts in aesthetic surgery, and the video roundtable was recorded live at The Aesthetic Meeting 2023 in Miami, FL, USA. The panelists describe their experiences using ultrasound in clinical procedures, the resulting contributions to safety in aesthetic surgery, its profound effect on patient experience, and ultimately its future as an indispensable tool in plastic surgery practices. Ultrasound has been available to plastic surgeons for years—in fact Dr. Brad Bengtson has utilized high-resolution ultrasound in his breast practice since the early 2000s.^1,2^ The use of ultrasound has become more prevalent and concerns about patient safety have brought the conversation to the national stage and news media outlets. In particular, the Brazilian Butt Lift (BBL), also known as gluteal augmentation, has been at the forefront of safety concerns in Miami, Florida. Several articles published in *Aesthetic Surgery Journal*^3^ and *ASJ Open Forum*^4,5^ contributed directly to improved outcomes and patient safety changes since ultrasound guidance provides sight into a previously blind procedure which offers unequivocal benefits.

Not only does ultrasound provide patients with a safer procedure, it offers surgeons additional anatomic precision. Plastic surgery advances when surgeons explore and learn more about anatomy and the use of ultrasound facilitates this education. Facial surgery procedures have also been advanced and improved with the use of ultrasound and discussant Dr. Chris Surek highlights examples during the discussion. The participants discussed the benefits of ultrasound, which led to a greater understanding of anatomy and navigating the danger zones. They commented that their improved sight during the procedure led to more natural clinical results.

The panelists agree that one of the most profound impacts of ultrasound has been its effect on the patient experience, not only in terms of safety and aesthetics, but an ineffable comfort that comes from complete trust in their surgeon and the procedure being performed. With ultrasound technology, patients can simultaneously observe what their surgeon sees, providing reassurance to patients who may have a lack of knowledge of general anatomy.

Finally, the panelists discuss the future of ultrasound and what comes next. The technology is rapidly advancing and will continue to do so with the help of artificial intelligence (AI). The hope is to combine 3-dimensional ultrasound with AI and create maps of the body, so surgeons can enter the operating room with a plan of the subcutaneous spaces in which they will be working, allowing them to prepare and visualize the surgery before it even begins.

## Supplementary Material

ojad060_Supplementary_Data
